# A Detached Gallbladder Situated Below the Transverse Colon: A Case Report of an Unusual Position

**DOI:** 10.7759/cureus.50245

**Published:** 2023-12-09

**Authors:** Moaz Abulfaraj

**Affiliations:** 1 Division of General Surgery, Department of Surgery, King Abdulaziz University Hospital, King Abdulaziz University, Jeddah, SAU

**Keywords:** floating gallbladder, acute surgical abdomen, laporoscopic cholecystectomy, congenital gallbladder abnormalities, acute calculus cholecystitis

## Abstract

This report presents an unforeseen event involving the detachment of the gallbladder without any traumatic cause, which was incidentally detected during a scheduled laparoscopic cholecystectomy procedure. The incidence of total congenital gallbladder detachment from the liver is quite uncommon. The primary difficulty encountered in patients afflicted with this particular medical issue pertains to the intraoperative identification and localization of the gallbladder. The significance of this case report lies in its presentation of a groundbreaking finding that has the potential to provide challenges for surgeons doing laparoscopic cholecystectomy, a commonly performed surgical procedure.

## Introduction

The surgical removal of the gallbladder is a commonly performed procedure [1], and a significant number of novel anatomical and pathological findings have been recorded [2-5]. Non-traumatic detachment of the gallbladder is a condition that entails the total separation of the gallbladder from the liver bed. However, apart from a case documented in 2009 [6], the author has not come across any other published reports regarding non-traumatic detachment of the gallbladder.

The current report is the second such report and provides a thorough analysis of an incident involving a detached gallbladder, which was incidentally observed during a scheduled laparoscopic cholecystectomy procedure. This case report is of notable importance and pertinence within the medical field, as it highlights the discovery of a non-traumatic gallbladder detachment during a planned laparoscopic cholecystectomy. This finding is particularly significant given the substantial volume of similar surgeries conducted annually.

## Case presentation

**Figure 1:**
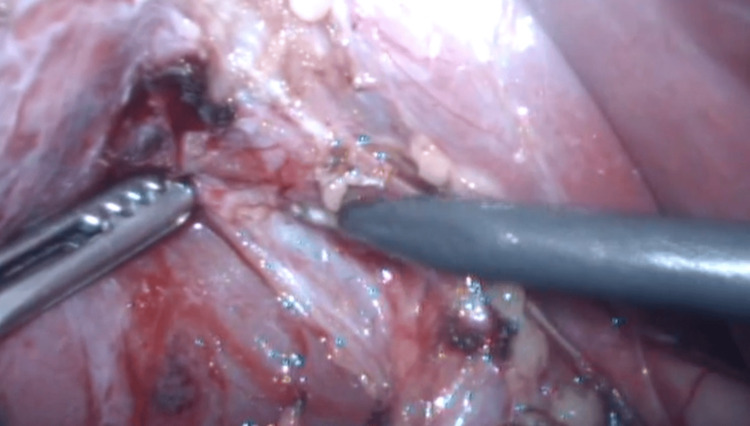
Empty gallbladder fossa

A female patient, aged 44 years, with a documented medical history of hypothyroidism, presented at the outpatient clinic with repeated instances of abdomen pain localized on the right side following meals. Approximately four years ago, the patient was diagnosed with gallstones following an ultrasound examination. Consequently, she underwent a laparoscopic intervention aimed at the removal of the gallbladder. However, the intraoperative exploration revealed the absence of the gallbladder within the hepatic parenchyma, resulting in the termination of the surgical intervention (Figure 1). The patient's symptoms continued to endure, leading to another laparoscopic endeavor aimed at identifying and extracting the gallbladder. Regrettably, this endeavor too proved futile.

**Figure 2:**
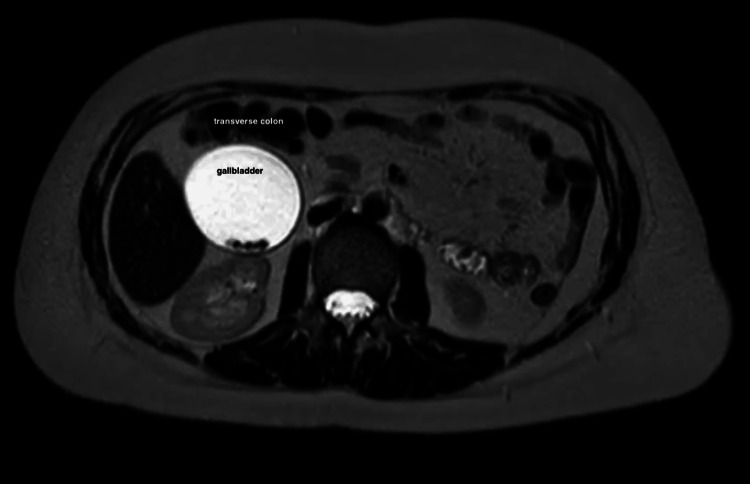
MRI showing the infracolonic location of the gallbladder

Due to the persistent nature of her pain, she decided to seek medical assistance at the outpatient clinic. Preliminary examinations, encompassing a comprehensive analysis of blood components and assessments of hepatic functionality, yielded unremarkable findings, characterized by a white blood cell count (WBC) of 7300 cells per cubic millimeter and a bilirubin concentration of 1.7 milligrams per deciliter. A transabdominal ultrasound examination was conducted, revealing the existence of numerous tiny stones that exhibited mobility. No indications of cholecystitis or any enlargement in the intra and extra-hepatic biliary system were observed. Additional preoperative assessment was conducted utilizing magnetic resonance imaging (MRI), which unveiled the presence of a gallbladder situated in a lower place within the abdomen, with the proximal transverse colon situated superior to it (Figure 2).

**Figure 3:**
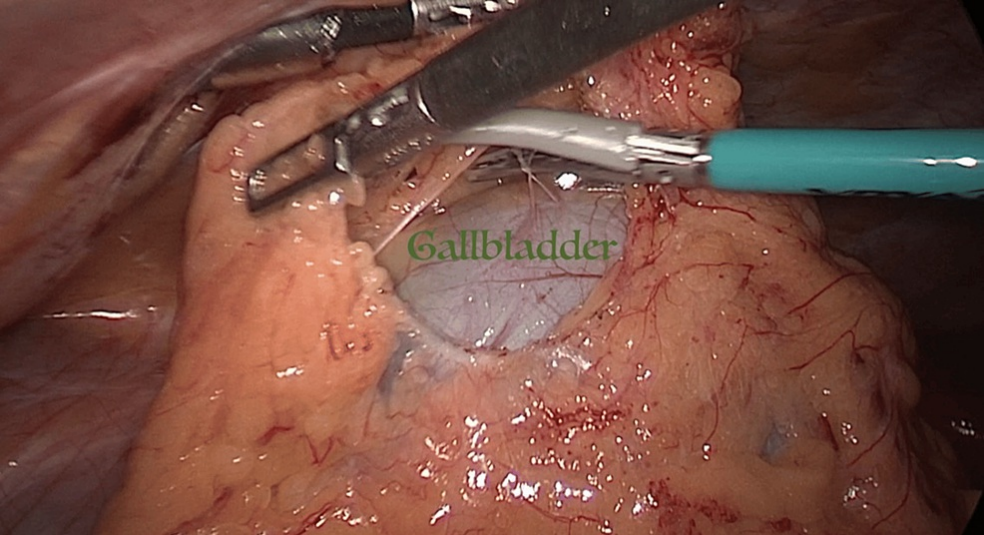
The gallbladder under the hepatocolic ligament

**Figure 4:**
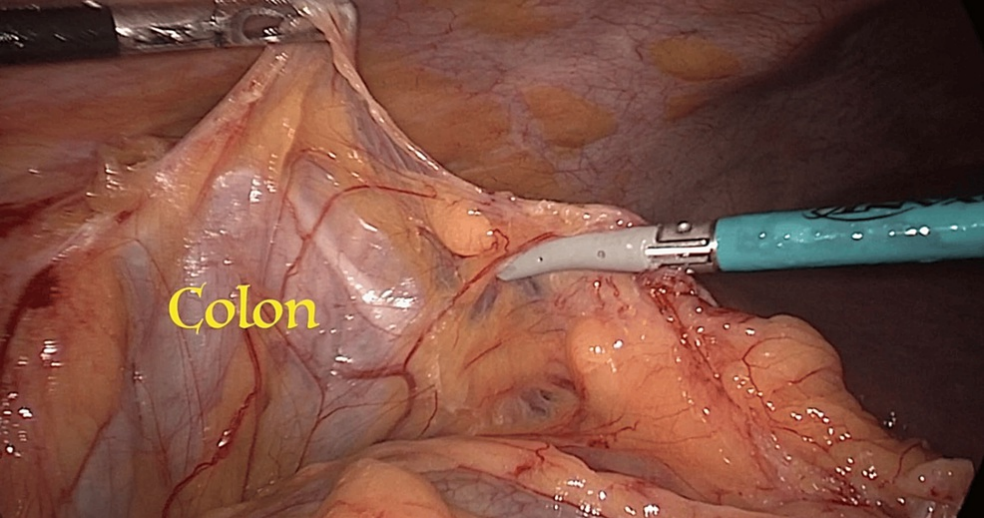
Downward retraction of the transverse colon

**Figure 5:**
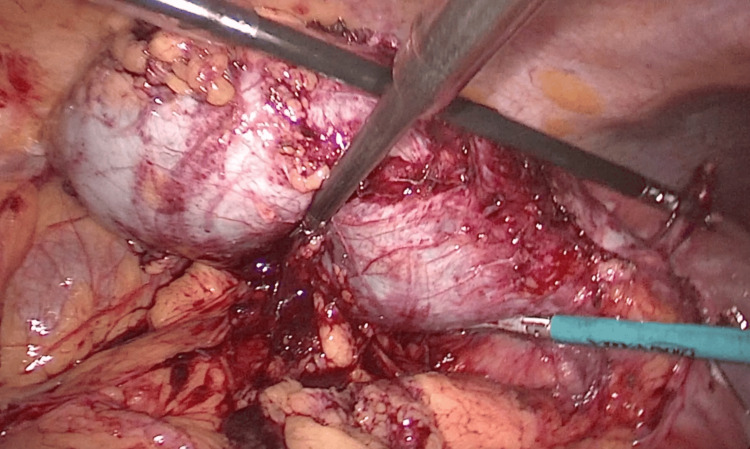
Freely mobile gallbladder

**Figure 6:**
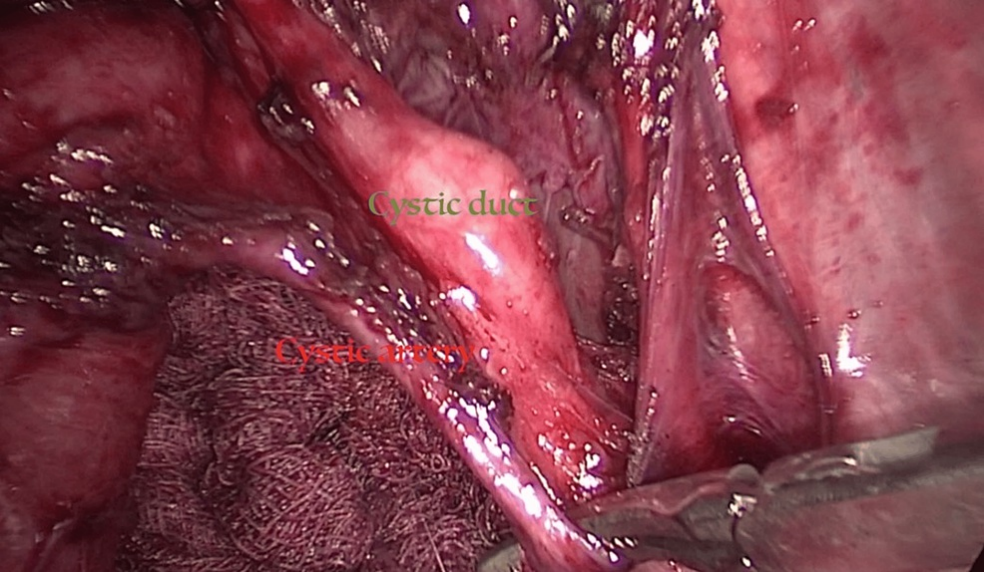
Separation between the cystic artery and duct

The patient underwent a surgical procedure in which the transverse colon was mobilized by splitting the larger omentum and subsequently retracted in a downward direction (Figure 3 and Figure 4). The gallbladder was promptly recognized and its origin was traced to the biliary tree. The liver was observed to be fully separated from its surrounding tissues, as depicted in Figure 5. The cystic duct and artery were readily discernible and successfully isolated from one another (Figure 6). Both structures underwent double clipping and cutting. The histopathological analysis of the excised gallbladder showed the existence of persistent inflammation and the presence of many sub-centimeter stones. Fortunately, there were no indications of cancer or necrosis observed.

After adhering to the established protocol, the patient experienced an uneventful postoperative recuperation, resulting in her subsequent discharge to her residence. During the four-week follow-up appointment, the patient said that she was no longer experiencing any symptoms.

## Discussion

Laparoscopic cholecystectomy is a prevalent surgical operation in the field of general surgery, with an annual occurrence of over 800,000 gallbladder removals in the United States [1]. While the procedure is commonly regarded as straightforward, there may be occasional difficulties that arise, especially in instances involving atypical anatomical structures. Instances of variations in the structure of the biliary tree and/or blood supply are frequently observed in these particular cases [2-5]. Nevertheless, it is crucial to acknowledge that occurrences of gallbladder placement changes are exceedingly uncommon. The solitary occurrence of congenital total detachment of the gallbladder from its typical location in the hepatic bed was documented in a singular instance in 2009 [6]. In the aforementioned example, the surgeon made the decision to alter the surgical operation to an open surgery, which ultimately resulted in the successful identification of the gallbladder in the infracolic region.

In the current case, prior to the present presentation, two surgeons made the decision to refrain from converting to an open surgery, despite the complexities associated with the atypical anatomical structure, ultimately discontinuing the process. It is important to acknowledge that the patient's thorough examination was not conducted until she visited the outpatient clinic. Fortunately, the utilization of magnetic resonance imaging (MRI) provided a clear depiction of the precise anatomical location of the gallbladder, which was shown to be situated posterior to the transverse colon. The utilization of preoperative imaging has enhanced the surgical technique, rendering it comparatively uncomplicated.

It is imperative to recognize that making intraoperative judgments might provide significant challenges in instances with atypical anatomical differences. The selection between open surgery, laparoscopic technique, or discontinuation of the process is contingent upon the surgeon's proficiency and evaluation of the circumstances. Often, there is no definitive or erroneous choice.

Conducting a thorough preoperative evaluation is of utmost importance, particularly in individuals with a medical history characterized by ambiguous anatomical observations. The application of cross-sectional imaging modalities, such as computed tomography (CT) scans or MRI, can significantly facilitate the accurate determination of the specific anatomical position of the gallbladder. Moreover, the utilization of magnetic resonance cholangiopancreatography (MRCP) can yield significant insights about the biliary tree.

A similar clinical manifestation to that of our patient is a disorder referred to as a floating gallbladder, which is also recognized as a wandering gallbladder. This atypical anatomical aberration manifests when the gallbladder exhibits unrestricted mobility or buoyancy as a result of extension of the connective tissue that ordinarily provides its anchorage. The symptoms attributed to a floating gallbladder may exhibit variability among individuals. Certain individuals may encounter persistent stomach pain or discomfort, although others may exhibit no symptoms. In instances of increased severity, the condition of a mobile gallbladder can lead to torsion and necrosis of the organ due to the twisting and subsequent obstruction of the cystic artery and duct [7-10].

## Conclusions

For individuals with atypical anatomical features, it is imperative to conduct a thorough preoperative assessment, which should encompass cross-sectional imaging techniques. Upon the identification of the anomaly and subsequent determination of the gallbladder's location, it is recommended that laparoscopic cholecystectomy be pursued as the preferable treatment modality. This approach is anticipated to be a rather uncomplicated procedure.
